# Langerhans cell histiocytosis in the adult lumbar spine: case report

**DOI:** 10.1186/s40064-016-3006-7

**Published:** 2016-08-23

**Authors:** Bobby Wirawan Hassan, Bong Ju Moon, Young-Jin Kim, Sang-Deok Kim, Ki-Young Choi, Jung-Kil Lee

**Affiliations:** Department of Neurosurgery, Chonnam National University Hospital and Medical School, 42, Jebong-ro, Donggu, Gwangju, 501-757 Republic of Korea

**Keywords:** Langerhans cell histiocytosis, Adult spine, Osteolytic lesion, Conservative method

## Abstract

**Introduction:**

Langerhans cell histiocytosis (LCH) occurs rarely in the spine of adults. The radiological findings usually resemble vertebral tumors. Etiology of LCH has not been clearly established yet. Therapeutic approaches are still controversial. We describe a case of LCH in an adult spine.

**Case description:**

A patient who presented with low back pain had an osteolytic lesion in the L1 vertebral body without neurological deficits, and fluoroscopy-guided needle biopsy of the L1 vertebral body was performed. The immunohistochemical diagnosis confirmed LCH. The patient was successfully treated with conservative methods.

**Discussion:**

The choice of appropriate therapy is very important, with treatment options varying from watch-and to aggressive treatment.

**Conclusion:**

LCH is considered as a pediatric disease that is extremely rare in the spine of adults and should be include in the differential diagnosis of osteolytic vertebral lesions. Conservative treatment is best choice for a patient with LCH without neurological deficit or spinal instability.

## Background

Langerhans cell histiocytosis (LCH) is a clonal proliferative disease characterized by infiltration of single or multiple organs by specific dendritic cells that resemble the normal epidermal Langerhans cell. LCH is considered a pediatric disease and is extremely rare in the spine of adults. Spinal involvement occurs in 6.5–25 % of all skeletal lesions. In the spine, LCH mainly involves the vertebral bodies, with a predilection for the thoracic spine (54 %) followed by the lumbar (35 %) and cervical spine (11 %) (Abu-Bonsrah et al. 2016). Only a small number of cases of LCH in the adult lumbar spine have been reported in the English literature (Huang et al. 2010; Demirci 2004; Bavbek et al. 2004; Bilge et al. 1995). This article describes the case of 35-year-old man diagnosed with LCH presenting as an osteolytic lesion of the L1 vertebral body.

## Case report

**Fig. 1:**
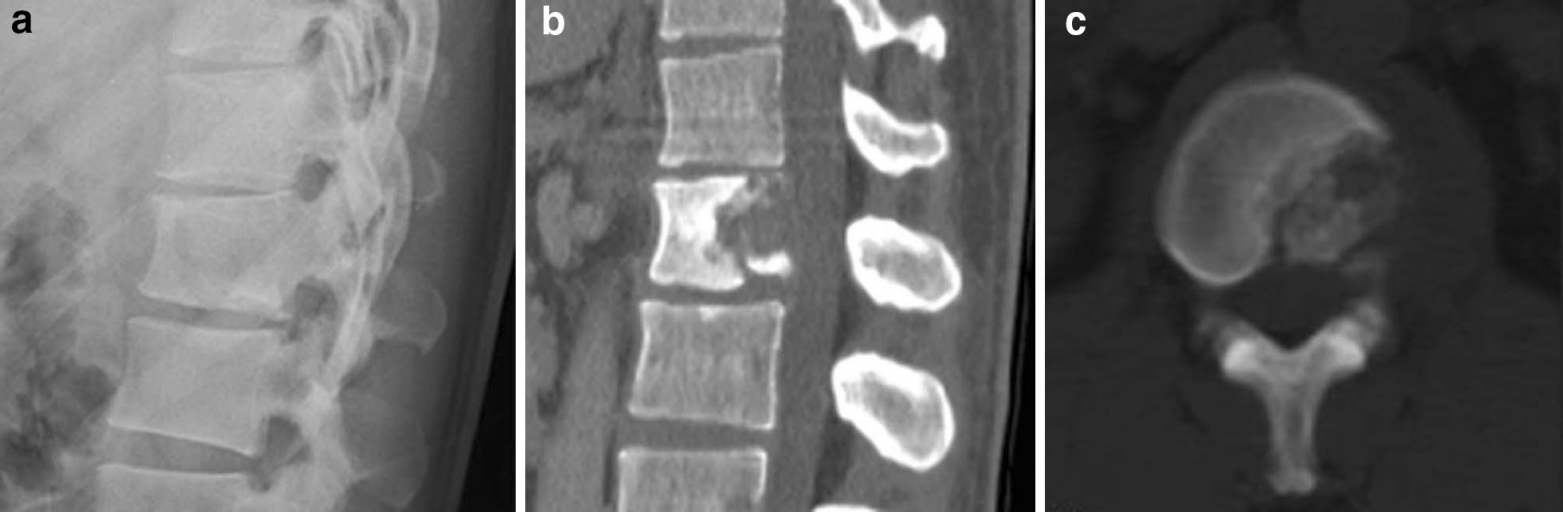
**a** Plain lumbar X-ray lateral view showing radiolucency of the L1 vertebral body. Sagittal (**b**) and axial (**c**) computed tomography showing an osteolytic lesion of the L1 vertebral body

**Fig. 2:**
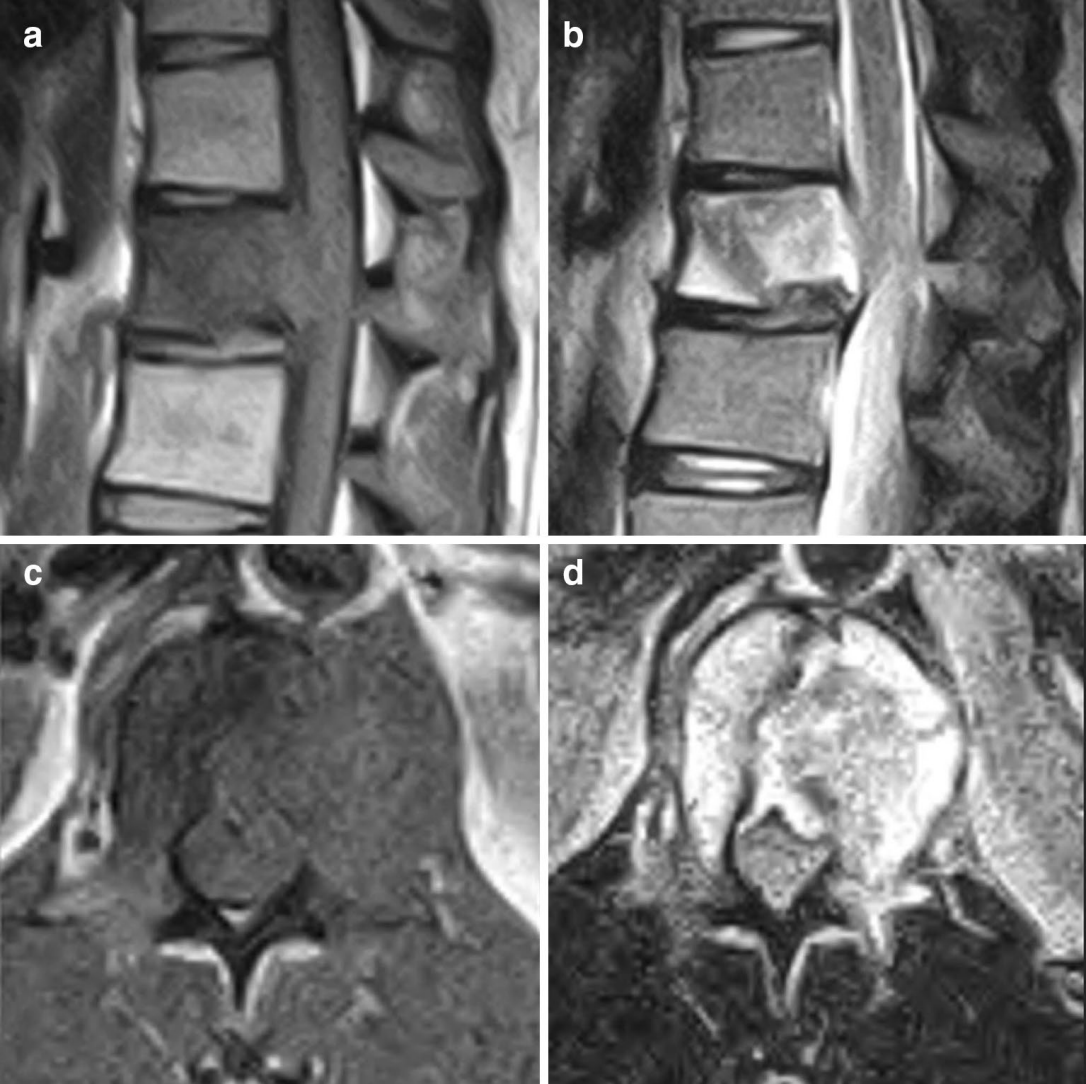
Sagittal magnetic resonance images showing low signal intensity changes of the L1 vertebral body on T1-weighted images (**a**) and high signal intensity changes on T2-weighted images (**b**). Axial magnetic resonance images showing low signal intensity changes of the L1 vertebral body and pedicle on T1-weighted images (**c**) and high signal intensity changes on T2-weighted images (**d**)

**Fig. 3:**
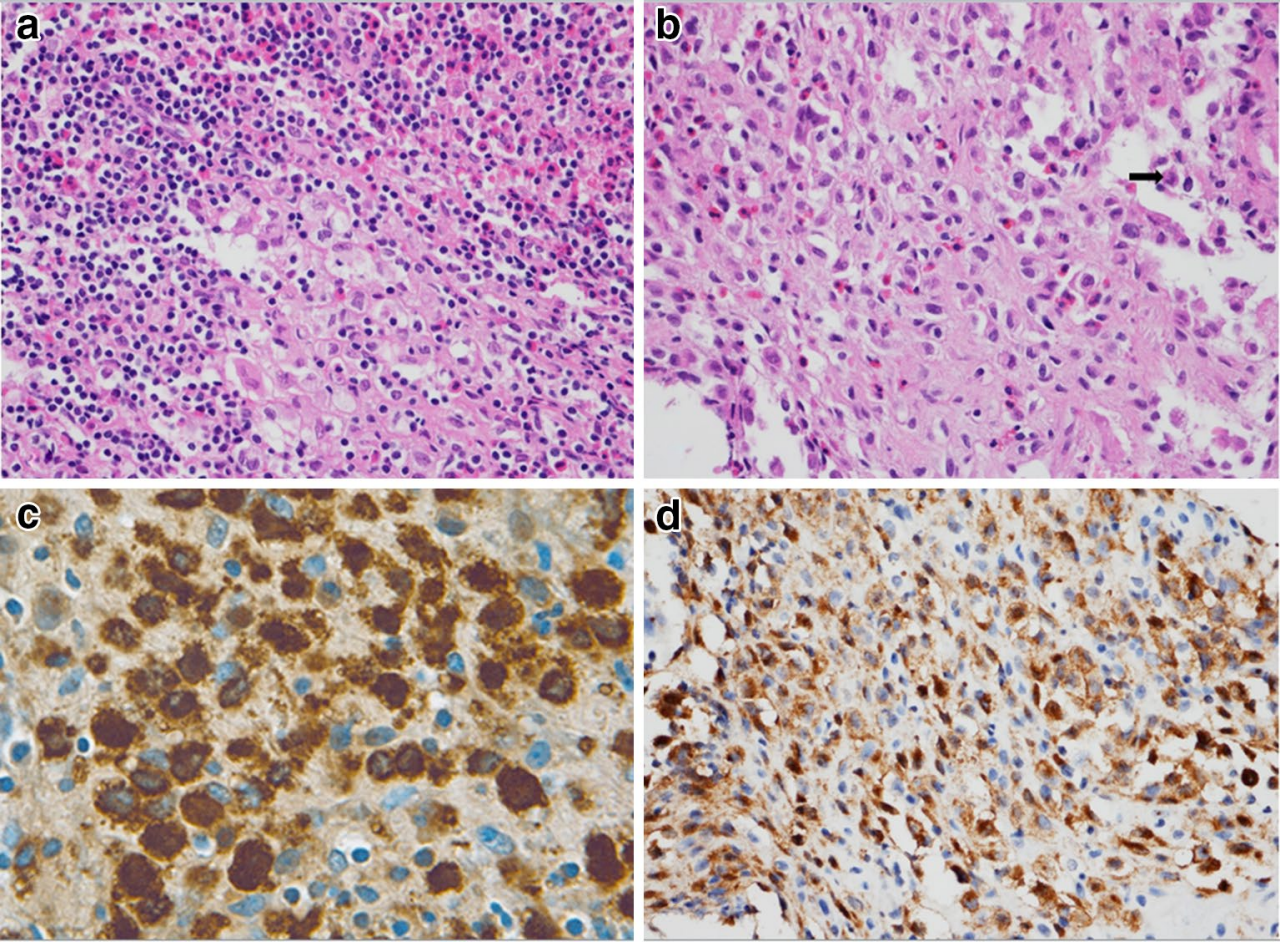
**a** High-power photomicrography showing Langerhans histiocytes and mixed numerous eosinophils. **b** Neoplastic cells showing folded and convoluted nuclei with intra-nuclear groove resembling coffee beans (*arrow*). Immunohistochemical staining for S-100 protein (**c**) and CD-1a (**d**) showing immunoreactivity of Langerhans cells

**Fig. 4:**
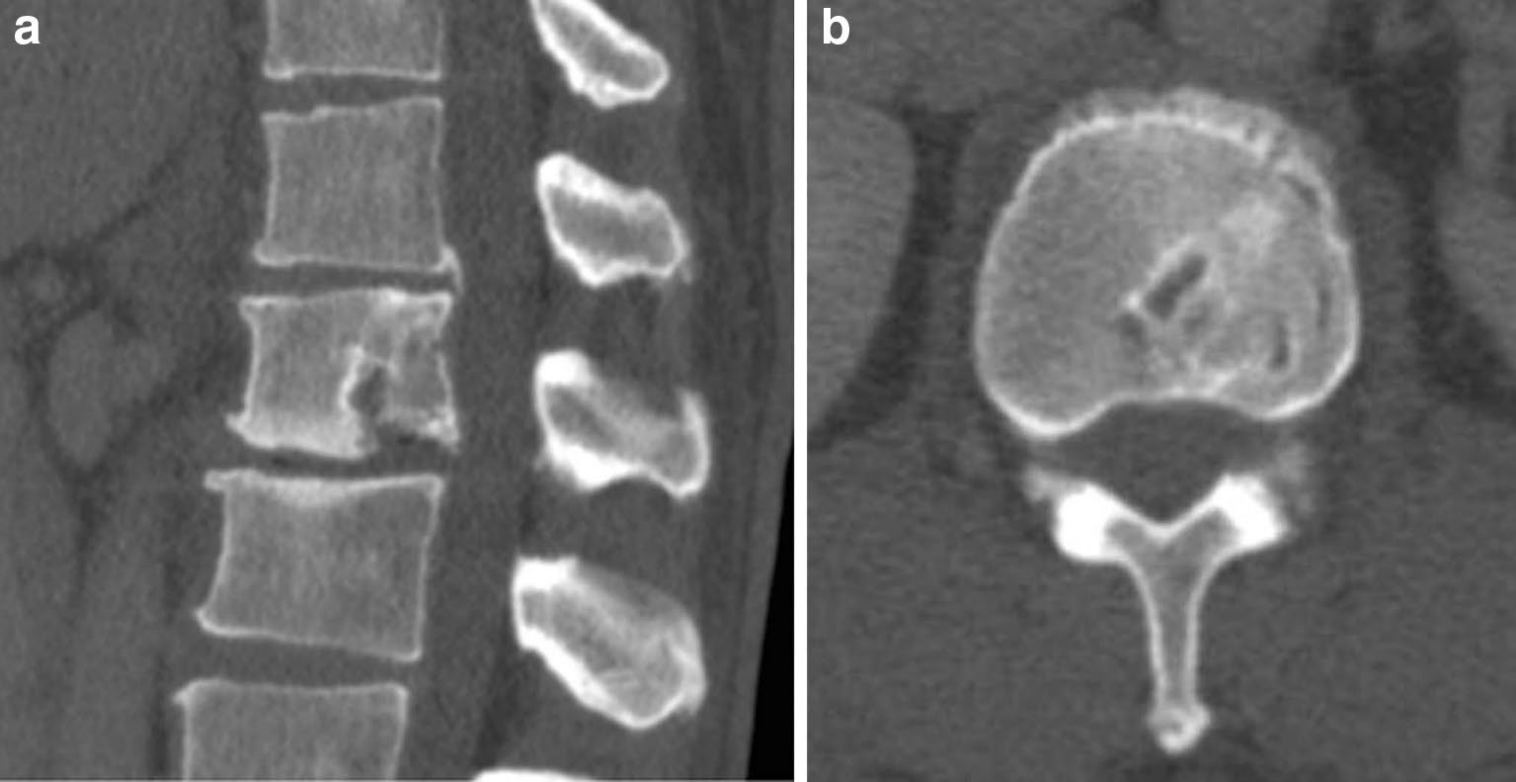
Sagittal (**a**) and axial (**b**) computed tomography a year after biopsy showing bone remodeling of the previous osteolytic lesion of the L1 vertebral body

A 35-year-old man presented with a one-year history of low back pain, and limited movement at the waist without any neurological deficit. He had no history of fever, night sweats, or weight loss, and was unable to recall any recent injury. The patient’s medical history was unremarkable for trauma or other bone diseases. A physical examination showed localized tenderness and percussive pain over the L1 spinous process, and restricted movement at the waist without any motor or sensory abnormalities. Laboratory tests including blood cell count, serum electrolytes, renal and liver function tests, erythrocyte sedimentation rate (ESR), and C-reactive protein (CRP) did not reveal any abnormalities. Plain lumbar spine X-ray revealed a radiolucency at the L1 vertebral body (Fig. 1a). Computed tomography (CT) showed a defined osteolytic lesion involving the left side of the vertebral body and pedicle of L1 (Fig. 1b, c). Magnetic resonance imaging (MRI) revealed low-signal intensity changes on T1-weighted images and high-signal intensity changes on T2-weighted images of the L1 vertebral body (Fig. 2). On the basis of the radiological features, there was a high possibility of a neoplastic lesion. A fluoroscopy-guided needle biopsy of the L1 vertebral body was performed. Histopathology showed proliferation of Langerhans cells arranged in sheets, with abundant eosinophilic cytoplasm and indentations on the nuclear membrane. Immunohistochemistry confirmed LCH with diffuse immunoreactivity of S-100 and CD1a (Fig. 3). No other lesion was found by bone scan. The patient was treated conservatively with nonsteroidal anti-inflammatory drugs and allowed to walk. 3 months after biopsy, bone remodeling of a destructive lesion of the L1 vertebral body was detected by CT. 2 years after biopsy, he was free from low back pain. Notably, follow-up CT revealed significant bone remodeling one year after biopsy, but the height of the vertebral body remained stable without further collapse, and lumbar kyphosis did not occur (Fig. 4). No evidence of recurrence or neurological deficit has been detected.

## Discussion

Langerhans cell histiocytosis (LCH) is a rare, clonal proliferative disease characterized by infiltration of single or multiple organs by specific dendritic cells that resemble the normal epidermal Langerhans cell. LCH comprises a spectrum of disorders. Localized disease is described as eosinophilic granuloma, while multisystem forms of LCH have eponymous descriptions such as Hand-Schüller-Christian disease, Letterer-Siwe disease, and systemic histiocytosis X. The present case was a solitary osseous lesion without systemic involvement. Although LCH most commonly occurs in the first two decades of life, it may affect patients of any age, from infants to elderly individuals. Among children, it has an annual incidence of 3-5 cases per million. In adults, the disease is rare, with an estimated prevalence of 1–2 cases per million/year (Azouz et al. 2005). Bone is the most common organ affected by LCH. The most frequent sites of the osseous lesions of LCH are the skull, femur, mandible, pelvis, and spine. LCH in the spine is reported to mainly involve the vertebral bodies, occurring in 6.5–25 % of cases, with the most frequent site being the thoracic vertebrae (54 %) in children, and the cervical vertebrae in adults. In the present case, LCH occurred in the lumbar vertebral body in an adult spine. Well-defined osteolytic lesions can be seen on radiological studies, although osteoblastic lesions can rarely develop (Beltran et al. 1993; Kilborn et al. 2003). In children, most of the vertebral body volume is infiltrated by LCH, causing complete collapse, with a characteristic “vertebra plana” appearance. In adults, a relatively small volume of the vertebral body is affected, and LCH of the body is present without collapse. CT can show some characteristic signs such as osteolytic lesions with a sclerotic margin. MRI can show a hypointense or heterogeneous signal intensity lesion on T1-weighted images and a hyperintense lesion on T2-weighted images. In the case of our patient, plain X-ray, CT, and MRI were performed, and an osteolytic lesion of the L1 vertebral body was identified. Although characteristic clinical and radiological features suggest the diagnosis, these features cannot provide a definitive diagnosis. Histological confirmation is always required in adults (Huang et al. 2010). Malignant tumor, osteomyelitis, aneurysmal bone cyst, Ewing’s sarcoma, osteoblastoma, Gaucher’s disease, and acute leukemia must be differentiated (Reddy et al. 2000). The diagnosis of LCH is confirmed by immunohistochemical staining for CD-1a and S-100 protein (Azouz et al. 2005). Spinal LCH is self-limiting and the prognosis is usually good. A variety of treatment modalities for spinal LCH have been reported, including conservative management, intralesional steroid injection, radiation therapy, chemotherapy, and curettage with or without reconstructive surgery (Puigdevall et al. 2008; Garg et al. 2003; Bertram et al. 2002). Mild, isolated involvement of the spine without neurological deficits or spinal instability can be managed with conservative methods such as simple observation, nonsteroidal anti-inflammatory drugs, immobilization, or casting with or without initial bed rest. Although radiation therapy has the potential for secondary malignancy and vertebral growth-plate damage in the skeletally immature patient, low-dose radiation therapy appears safe and effective for adult patients with progressive spinal lesions or neurological impairment. Chemotherapy, especially modest dose of ARA-C (100 mg/m^2^ daily for 5 days, repeated monthly for 6) is suggested for disseminated LCH with multiple bone lesions or multisystem disease (Cantu et al. 2012). Recent studies have been reported that administration of pegylated interferon-alpha can be helpful for treatment of LCH by inducing anti-tumoral response (Furudate et al. 2014). A large fraction of LCH cases appear to be associated with BRAF mutations and those could potentially benefit from treatment with a BRAF inhibitor (Badalian-Very et al. 2010). Surgery should be reserved for selected cases with severe mechanical instability, deformity, or neurological deficit due to compressionBecause spinal LCH is self-limiting, most authors have recommended conservative treatment for disease without neurological or mechanical complications, as in our case.

## Conclusion

LCH is considered a pediatric disease that is extremely rare in the spine of adults. However, it should be included in the differential diagnosis of osteolytic vertebral lesions in adults. Conservative treatment is a good choice for a patient with LCH without neurological deficits or spinal instability.
